# International Expert Consensus Statement on Chronic Endometritis: A Comprehensive Literature Review and Modified e‐Delphi Study

**DOI:** 10.1002/rmb2.70066

**Published:** 2026-06-12

**Authors:** Kotaro Kitaya, Keiji Kuroda, Seung Chik Jwa, Michio Kitajima, Masanori Ono, Iwaho Kikuchi, Tomoko Nakamura, Fumio Yamasaki, Kaori Koga, Tadahiro Yasuo, Shunichiro Tsuji, Fuminori Kimura, Ayumu Ito, Yasushi Hirota, Mari Nomiyama, Nanako Iwami, Kanako Takimoto, Isao Takehara, Yusuke Sako, Kuniaki Ota, Lianghui Diao, Yuye Li, Lei Yan, Wei Huang, Shen Zhang, Qiong Wang, Fang Gu, Jing Shu, Mahvash Zargar, Vitaly A. Kushnir, Claudio Benadiva, Constantin‐Cristian Văduva, Brecht Geysenbergh, Orestis Tsonis, Tirso Pérez‐Medina, Inmaculada Moreno, Sergio Haimovich, Amerigo Vitagliano, Ettore Cicinelli, Maki Kusumi

**Affiliations:** ^1^ The Japan Society of Reproductive Medicine Female Reproductive Tract Special Interest Group on Chronic Endometritis Tokyo Japan; ^2^ Infertility Center Iryouhoujin Kouseikai Mihara Hospital/Katsura‐Ekimae Mihara Clinic Kyoto Japan; ^3^ Center for Reproductive Medicine and Endoscopy Sugiyama Clinic Marunouchi Tokyo Japan; ^4^ Department of Obstetrics and Gynecology Juntendo University Faculty of Medicine Tokyo Japan; ^5^ Department of Obstetrics and Gynecology Jichi Medical University Tochigi Japan; ^6^ Women's Medical Center Takagi Hospital Okawa Japan; ^7^ Department of Obstetrics and Gynecology International University of Health and Welfare Okawa Japan; ^8^ Department of Obstetrics and Gynecology Keio University School of Medicine Tokyo Japan; ^9^ Medical Park Yokohama Yokohama Japan; ^10^ Department of Obstetrics and Gynecology Nagoya University Graduate School of Medicine Nagoya Japan; ^11^ Department of Pathology Japan Community Health Care Organization, Saga Central Hospital Saga Japan; ^12^ Department of Gynecology Chiba University Hospital Chiba Japan; ^13^ Department of Reproductive Medicine Chiba University Chiba Japan; ^14^ Department of Obstetrics and Gynecology Otsu City Hospital Otsu Japan; ^15^ Department of Obstetrics and Gynecology Shiga University of Medical Science Otsu Japan; ^16^ Department of Obstetrics and Gynecology Nara Medical University Kashihara Japan; ^17^ Department of Obstetrics and Gynecology Faculty of Medicine, Toho University Tokyo Japan; ^18^ Department of Obstetrics and Gynecology Graduate School of Medicine, The University of Tokyo Tokyo Japan; ^19^ Kamiya Ladies Clinic Sapporo Japan; ^20^ Department of Obstetrics and Gynecology Faculty of Medicine, Yamagata University Yamagata Japan; ^21^ Department of Obstetrics and Gynecology St. Luke's International Hospital Tokyo Japan; ^22^ Department of Obstetrics and Gynecology Kawasaki Medical School Kurashiki Japan; ^23^ Shenzhen Key Laboratory of Reproductive Immunology for Peri‐Implantation, Shenzhen Zhongshan Institute for Reproductive Medicine and Genetics, Shenzhen Zhongshan Obstetrics and Gynecology Hospital Shenzhen China; ^24^ Center for Reproductive Medicine, Cheeloo College of Medicine, Shandong University Jinan Shandong China; ^25^ Division of Reproductive Medicine West China Second University Hospital of Sichuan University Chengdu China; ^26^ Laboratory of Clinical Embryology, Reproductive Medicine Center, Department of Obstetrics and Gynecology The Second Affiliated Hospital, Chongqing Medical University Chongqing China; ^27^ Department of Obstetrics and Gynecology Reproductive Medical Center, First Affiliated Hospital of Sun Yat‐Sen University Guangzhou China; ^28^ Guangdong Provincial Clinical Research Center for Obstetrical and Gynecological Diseases Guangzhou China; ^29^ Reproductive Medical Center, The First Affiliated Hospital, Zhejiang University School of Medicine Hangzhou Zhejiang China; ^30^ Fertility, Infertility and Perinatology Research Center, Department of Obstetrics and Gynecology Ahvaz Jundishapur University of Medical Sciences Ahvaz Iran; ^31^ Department of Obstetrics and Gynecology The University of California Irvine California USA; ^32^ Center for Advanced Reproductive Services Farmington Connecticut USA; ^33^ Department of Obstetrics and Gynecology Faculty of Medicine, University of Medicine and Pharmacy of Craiova Craiova Romania; ^34^ ZAS Augustinus Oosterveldlaan Wilrijk Belgium; ^35^ Assisted Conception Unit, Guy's Hospital, Guy's and St Thomas' NHS Foundation Trust London UK; ^36^ Hospital Universitario Puerta de Hierro Majadahonda Madrid Spain; ^37^ Carlos Simon Foundation, Valencia, Spain; INCLIVA Biomedical Research Institute Valencia Spain; ^38^ Department of Obstetrics and Gynecology at The Adelson Med School Ariel University Ariel Israel; ^39^ Unit of Obstetrics and Gynaecology, Department of Interdisciplinary Medicine University of Bari “Aldo Moro”, Policlinico of Bari Bari Italy; ^40^ Center for Human Reproduction and Gynecologic Endoscopy, Sanno Hospital Tokyo Japan

## Abstract

**Purpose:**

This study aimed to develop international expert consensus statements on chronic endometritis (CE) through a comprehensive literature review of existing evidence and a modified Delphi approach.

**Methods:**

Ten panelists of the Japan Society of Reproductive Medicine Female Reproductive Tract Special Interest Group on CE performed a comprehensive review of the literature and derived statements with related comments on six specific domains of interest on CE. A two‐round modified e‐Delphi questionnaire was conducted among 31 international experts to evaluate the statements.

**Results:**

Twenty‐three out of 24 statements, including 43 detailed items, on epidemiology (associated diseases and risk factors), symptomatology (asymptomatic or oligosymptomatic nature with subtle and nondescript gynecologic manifestations), etiology and pathogenesis (inflammation, infection, and clinical course), microbiology (associated pathogens), diagnosis (histopathology, immunohistochemistry, hysteroscopy, and microbiome analysis), and treatment (antibiotics, surgery, and multidrug resistance) finally reached a consensus. Notably, for the histopathologic diagnosis of CE, a threshold of ≥ 5 endometrial stromal plasma cells/10 high‐power fields received an agreement rate of 81%.

**Conclusions:**

This comprehensive literature review and Delphi study provide a real‐world clinical perspective on CE from diverse international experts. These consensus statements have the potential to lay a foundation for diagnostic criteria and/or clinical guidelines on CE.

## 1 Introduction

Chronic endometritis (CE) is a localized inflammatory disorder recognized as the unusual infiltration of endometrial stromal plasma cells (ESPCs) [1, 2]. Local infection is considered to be the primary cause of CE, as antibiotic treatment has been reported to be effective in eradicating ESPCs and potentially improving the pregnancy outcomes in the subsequent treatment cycles of infertile women [3, 4]. Increasing attention has been directed toward CE over the past decade, particularly because of its potential involvement in reproductive failure, including recurrent implantation failure (RIF) and recurrent pregnancy loss (RPL) [5, 6, 7]. In 2023, the European Society of Human Reproduction and Embryology Working Group published Good Practice Recommendations on RIF, in which assessment for CE can be considered for infertile women suffering from RIF following in vitro fertilization–embryo transfer programs, and antibiotic treatment can be considered if CE is diagnosed in these patients [8].

The diagnosis of CE, however, remains challenging. Lack of specific and characteristic symptoms hinders the diagnosis of CE [2, 6]. Histopathology has been the traditional diagnostic method for this disease [1]. Despite the development of immunohistochemical detection of ESPCs [9, 10, 11], several concerns have been raised in their clinical application. These include (i) misidentification of endometrial epithelial cells, which constitutively express a plasma cell marker CD138, as ESPCs, potentially leading to the overdiagnosis of CE; (ii) sampling method, device, and phases for endometrial biopsy that can affect the diagnostic performance of CE, as ESPCs tend to appear as focal infiltrates rather than distributing evenly; (iii) optimal threshold for ESPC density that has not yet been defined for the histopathologic diagnosis of CE, particularly for infertile women, as a few ESPCs are found in 30% of healthy fertile women [12, 13]. Fluid hysteroscopy and microbiome analysis are emerging as alternative diagnostic tools for CE. Studies, however, are insufficient to validate their consistency with histopathologic examinations [14, 15, 16, 17, 18]. The optimal antibiotic agents and regimens also remain undetermined. Although broad‐spectrum oral doxycycline has been preferentially administered for the treatment of CE due to its convenience, safety, and effectiveness against pelvic inflammatory diseases, few studies have demonstrated its superiority over other antibiotics [19].

Thus, to date, no definitive diagnostic criteria and/or clinical guidelines exist for CE. The establishment of the unified clinical framework for CE is strongly desired to enhance diagnostic accuracy, inform treatment decisions, and ultimately improve reproductive outcomes. The Delphi method is used to generate a reliable consensus opinion by a group of experts through an iterative process of questionnaires interspersed with controlled feedback [20]. The Delphi method has been widely adopted in healthcare, medical research, and the development of clinical practice [21]. Through a comprehensive literature review of existing evidence by panelists and a modified e‐Delphi approach, this study aimed to develop international expert consensus statements, laying a foundation for diagnostic criteria and/or clinical guidelines on CE.

## 2 Methods

### 2.1 Outline

**FIGURE 1:**
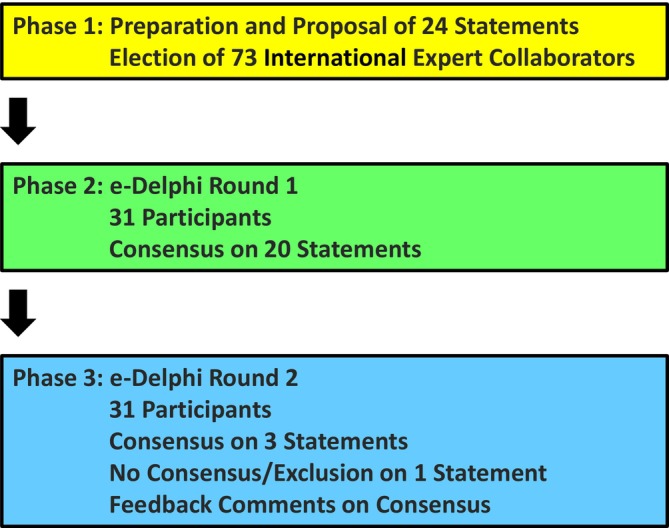
Design of the e‐Delphi consensus study.

The Japan Society of Reproductive Medicine Female Reproductive Tract Special Interest Group (SIG) on CE was formed on November 15, 2024, comprising 10 panelists: eight reproductive endocrinologists/obstetricians/gynecologists; one reproductive endocrinologist/biostatistician; and one clinical pathologist. SIG first conducted an electronic database search for literature and held web meetings to determine the inclusion/exclusion criteria, discuss six specific domains of interest on CE, including (1) epidemiology, (2) symptomatology, (3) etiology and pathogenesis, (4) microbiology, (5) diagnosis, and (6) treatment, and performed a comprehensive review of the available literature. No formal review protocol was prepared or registered. SIG extracted consensus statements and related comments on CE (Figure 1), adopted a modified e‐Delphi method, and designed a questionnaire platform to effectively solicit responses from international experts on CE.

### 2.2 Electronic Database Search and Inclusion/Exclusion Criteria of Literature

**FIGURE 2:**
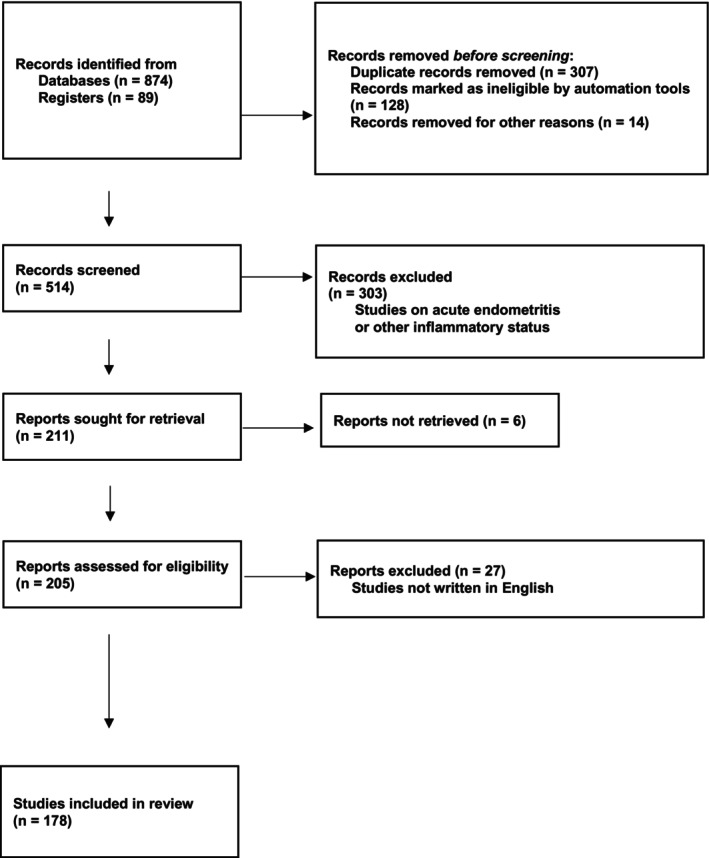
Flow diagram of database search and study inclusion and exclusion process.

PubMed was used as the primary electronic database, and Google Scholar as the supplementary database. Both databases were searched for articles from their inception to March 31, 2025. The final search date was April 4, 2025. SIG used only the search terms associated with the target condition to maximize search sensitivity. The following PubMed search strategy was used to identify relevant studies on chronic endometritis. The Boolean operators (i.e., “chronic endometritis” OR “endometritis” OR “subclinical pelvic inflammatory disease” OR “endometrial inflammation” [Title/Abstract]) and Medical Subject Headings “endometritis” were adopted. Filters were applied for humans, the English language, and full text available. The study types included randomized controlled trials, observational studies (prospective or retrospective cohort studies and case–control studies), review articles, case series, and case reports. Additional inclusion criteria were a clear description of the diagnostic criteria for CE in the text and the use of histopathologic, hysteroscopic, and microbial examinations as reference standards. Conference and meeting abstracts were excluded from the study. Google Scholar was searched using the following keywords: “chronic endometritis.” Due to its limited reproducibility, relevance was used consistently for sorting, and the first 500 records were screened based on the results of the primary electronic database screening. Embase was not included in this study because, in a previous review of CE, most (> 99%) of its CE search results overlapped with those of PubMed and did not yield results superior to PubMed [6]. The Cochrane Central Register of Controlled Trials was searched, and no additional associated articles were found. Titles and abstracts of the literature were independently screened by two authors (K.Ku. & S.C.J.) to assess study features (design, country, and timeline), populations (number of participants and characteristics), diagnostic criteria, and study outcomes. The inter‐author disagreement was resolved by discussion. A manual search of the included literature for references was conducted to avoid missing relevant data. When details were not available from the text, tables, figures, and/or Supporting Information, the authors of the study in question were contacted by email, if applicable. The process of record identification and screening, and the reports sought for retrieval and assessed for eligibility, is summarized in Figure 2.

### 2.3 Comprehensive Literature Review and Focus Group Discussion

In advance of literature review, SIG discussed the following variables of relevance: study design (objectives, inclusion and exclusion criteria, definition of CE, number of patients allocated to intervention group and control group, follow‐up time, sample size estimations, sponsorships, and conflict of interest statements and trial registry identifiers [if applicable]), patient demographic‐related variables (age, diagnosis or treatment‐related data, indications for treatments, and treatments administered), study methodology (information on sequence generation, allocation concealment, degree and success of blinding for randomized controlled studies, data completeness, missing data handling and possible effects, and intention‐to‐treat analysis), and selective report on other sources of bias (dissimilarity of allocated groups, cointerventions not distributed evenly among the groups, compliance differences, and differences in timing of the outcome assessment). SIG conducted a literature review and a focus group discussion between April 2025 and August 2025, and derived 24 consensus statements with related comments between September 2025 and November 2025. These statements and comments were based on existing evidence in the literature; thus, institutional approval was not required. Following the risk of bias assessment, all members consented to their inclusion in this manuscript.

### 2.4 International Expert Recruitment and Modified e‐Delphi Rounds

With reference to a bibliometric analysis study published in May 2025 [22], SIG listed 73 candidate international experts, all of whom had a history of (i) two or more publications over the past ten years or (ii) one or more publications over the past three years on CE in peer‐reviewed journals. They were invited to participate in the study and complete the questionnaire in November 2025. All participants provided written informed consent. In the e‐Delphi round 1, the participants were asked to evaluate each statement using a 3‐point Likert scale (agree, neutral, or disagree). The following definitions were used: Agree: “I agree with the statement,” Neutral: “I do not have an opinion about the statement”, and Disagree: “I do not agree with the statement.” Additional clarifying questions were included if required. The space for reflections about the statements was provided at the end of the questionnaire. When the consensus on the particular statement was not reached, the statement was reworded, reformulated, and carried over to Round 2, in which the participants were asked to reevaluate the revised statements again using a 3‐point Likert scale. As previously described [23, 24], “Reach a Consensus” was defined as [= 100 (agreement)/(agreement + neutral + disagreement)] or disagreement rate [= 100 (disagreement)/ (agreement + neutral + disagreement)] of 70% or greater of valid responses. The statements that did not reach a consensus were excluded.

## 3 Results

### 3.1 Overview

Thirty‐one experts who met the inclusion criteria agreed to participate in the study. Their demographic characteristics are shown in Table 1.

**TABLE 1:** Demographic characteristics of the experts on CE who participated in the e‐Delphi questionnaire.

Characteristics	
Location	
East Asia	19 (61%)
Europe	9 (29%)
Middle‐East	1 (3%)
North America	2 (6%)
Type of Professionals	
Basic researchers	4 (13%)
Obstetrician/gynecologist (including reproductive endocrinologist)	27 (87%)
Affiliation	
Private facility	10 (32%)
Public facility	2 (6%)
University	19 (61%)

In Round 1, 40 out of 67 detailed items in 24 statements reached a consensus with agreement. There were no detailed items that reached a consensus with disagreement. Fourteen statements (Statements 3A, 3B, 3D, 4B, 5A, 5B, 5C, 5D, 5E, 5G, 5H, 5I, 6A, 6B) reached a consensus with agreement on all items. Six statements (Statements 1A, 1C, 3C, 4A, 5F, 6C) reached a consensus with agreement after the exclusion of 22 items that did not reach a consensus and reformulation/rewording of the statements. Three statements (Statements 1B, 2, and 6D) did not reach a consensus and were reformulated/reworded/carried over to Round 2, where they reached a consensus with agreement. The Statement “Viral infections, such as herpes viruses and human immunodeficiency virus, could be potential pathogens for CE.” did not reach a consensus in Round 1, with less than one‐third agreement for both items: herpes viruses—agreement rate 32.3% (agree, 10/31; neutral, 11/31; disagree, 10/31) and human immunodeficiency virus—agreement rate 22.6% (agree, 7/31; neutral, 15/31; disagree, 9/31), and were excluded from the study.

Ultimately, 23 out of 24 statements reached a consensus with agreement. The statements that reached a consensus with agreement and comments are as follows: (Table 2).

**TABLE 2:** International expert consensus statements on CE, domains 1–3 (epidemiology, symptomatology, and etiology and pathogenesis), domains 4 and 5 (microbiology and diagnosis), and domains 5 and 6 (diagnosis and treatment).

Statements	Agreement rate
*Domain 1 (epidemiology)*	
1A: CE‐associated diseases include fallopian tubal infertility (hydrosalpinx and tubal occlusion), endometrial polyps, intrauterine adhesion/Asherman syndrome, and cesarean scar disorder. CE is also involved in unexplained infertility, recurrent implantation failure in in vitro fertilization–embryo transfer cycles, and unexplained recurrent pregnancy loss. Meanwhile, the causality between CE and female infertility or pregnancy loss remains undetermined	
Fallopian tubal infertility (hydrosalpinx and tubal occlusion)	74%
Endometrial polyps	87%
Intrauterine adhesion/Asherman syndrome	74%
Cesarean scar disorder	81%
Unexplained infertility	87%
Recurrent implantation failure	97%
Recurrent pregnancy loss	94%
1B: Endometrial osseous metaplasia is a rare disease associated with CE and infertility	71%
1C: Several risk factors have been proposed regarding the onset of CE. A history of continuous intrauterine contraceptive device use, prolonged menstruation, atypical uterine bleeding, and pregnancy loss (miscarriage) are the independent risk factors for CE	
Intrauterine contraceptive device use	71%
Prolonged menstruation	71%
Atypical uterine bleeding	74%
Pregnancy loss	81%
*Domain 2 (symptomatology)*	
CE is generally asymptomatic or oligosymptomatic with subtle and nondescript gynecologic manifestations	84%
*Domain 3 (etiology and pathogenesis)*	
3A: CE is a localized inflammatory disorder of the endometrium that is histopathologically characterized by superficial mucosal edema, increased endometrial stromal cell density, unsynchronized differentiation between endometrial epithelial cells and stromal cells, and unusual infiltration of endometrial stromal plasma cells. Of these histopathologic features, the most specific and sensitive findings of CE are thought to be the presence of multiple stromal plasma cells	90%
3B: The etiology and pathogenesis of CE, including its onset, progress, and remission, remain unclear. Endometrial shedding during the menstrual period is unlikely to contribute significantly to the treatment of CE. In some cases, CE seems to recur following treatment	94%
3C: Chronic deciduitis is potentially a persistent form of CE during pregnancy and is associated with preterm labor	71%
3D: One of the major causes of CE is microbial infection in the uterine cavity. This is supported by the fact that some antibiotic therapies are effective in eliminating endometrial stromal plasma cells in women with CE	90%
*Domain 4 (microbiology)*	
4A: The microorganisms detected in the uterine cavity of women with CE include common bacteria, such as *Streptococcus*, *Escherichia coli* , *Enterococcus faecalis* , and *Staphylococcus* species, as well as *Mycoplasma*/*Ureaplasma* species	
*Streptococcus*	74%
*Escherichia coli*	77%
*Enterococcus faecalis*	71%
*Staphylococcus* species	71%
*Mycoplasma* species	74%
*Ureaplasma* species	77%
4B: *Mycobacterium tuberculosis* is a microorganism that causes chronic granulomatous endometritis, a subtype of CE characterized by poorly developed caseating granuloma and surrounding lymphocyte infiltrates, including stromal plasma cells	74%
*Domain 5 (diagnosis)*	
5A: Endometrial biopsies are recommended to obtain the specimens for histopathologic diagnosis of CE in infertile women. Endometrial samplings by smear or brushing are not recommended	94%
5B: The addition of immunohistochemistry for CD138, a plasma cell marker, improves sensitivity, specificity, interobserver variability, and intraobserver variability compared with the conventional tissue staining alone in the histopathologic diagnosis of CE	90%
5C: Optimized laboratory test settings and quality control of CD138 immunostaining, including antibody selection and dilution, incubation time, thickness of endometrial sections, and number and area of sections, are indispensable for accurate diagnosis of CE	94%
5D: Caution should be exercised in the interpretation of the results of immunohistochemistry in CE, as not only endometrial stromal plasma cells, but endometrial epithelial cells, constitutively express CD138. The conditions of the section preparation and/or staining intensity may cause a mix‐up between these two cell types, potentially leading to the overdiagnosis of CE	81%
5E: The detection rate of endometrial stromal plasma cells in CE is higher in the proliferative phase of the menstrual cycle than in the secretory phase	81%
5F: The solid threshold of endometrial stromal plasma cell density and the number of microscopic fields to be observed for identification of endometrial stromal plasma cells remain undetermined for histopathologic diagnosis of CE. Given the reproductive outcomes in the following infertility treatment cycles, a threshold of ≥ 5 plasma cells/10 high power fields (≥ 0.5 plasma cells/high power field) may be appropriate for the histopathologic diagnosis of CE	81%
5G: Fluid hysteroscopy emerges as an alternate minimally invasive diagnostic modality for CE. Five hysteroscopic features suggestive of CE are proposed by the Delphi consensus method. These include (1) strawberry aspect: large areas of hyperemic endometrium flushed with white central points, (2) focal hyperemia: small areas of hyperemic endometrium; (3) hemorrhagic spots: focal red areas with sharp and irregular borders possibly in continuity with capillary; (4) endometrial micropolyps: small intrauterine new growths < 1 mm in size with a distinct connective vascular axis, distributed on focal areas or the entire endometrial surface; and (5) endometrial edema in the follicular phase: thick and pale appearance of the endometrium owing to stromal edema (a normal finding during the secretory phase)	
Strawberry aspect	97%
Focal hyperemia	84%
Hemorrhagic spots	81%
Endometrial micropolyps	94%
Endometrial edemas	71%
5H: There is substantial incompatibility between histopathologic and hysteroscopic diagnosis of CE. In the current clinical practice, fluid hysteroscopy alone is insufficient for the accurate diagnosis of CE	74%
5I: Recent progress in molecular microbiology (next‐generation sequencing testing of the 16S rRNA targeted gene or real‐time polymerase chain reaction for the causal pathogens of CE) enabled the comprehensive analysis of culturable and non‐culturable pathogens associated with CE	77%
*Domain 6 (treatment)*	
6A: If CE is diagnosed in women with a history of recurrent implantation failure, antibiotic treatment can improve subsequent pregnancy outcomes	84%
6B: If CE is diagnosed in women with a history of recurrent pregnancy loss, antibiotic treatment can improve subsequent pregnancy outcomes	71%
6C: Surgical treatment may be prioritized in CE that is concomitant with endometrial polyps, intrauterine adhesions/Asherman syndrome, and hydrosalpinx	
Endometrial polyps	87%
Intrauterine adhesions	87%
Hydrosalpinx	81%
6D: Antibiotic resistance is a serious global problem in the treatment of infectious diseases. Surveillance of emerging multidrug‐resistant CE is necessary	90%

### 3.2 Domain 1 (Epidemiology)

Statement 1A *CE‐associated diseases include fallopian tubal infertility (hydrosalpinx and tubal occlusion), endometrial polyps, intrauterine adhesion/Asherman syndrome, and cesarean scar disorder. CE is also involved in unexplained infertility, recurrent implantation failure in in vitro fertilization–embryo transfer cycles, and unexplained recurrent pregnancy loss. Meanwhile, the causality between CE and female infertility or pregnancy loss remains undetermined*.

This statement was reworded and reformulated from “CE‐associated diseases include endometriosis, fallopian tubal infertility (hydrosalpinx and tubal occlusion), polycystic ovarian syndrome, endometrial polyps, intrauterine adhesion/Asherman syndrome, cesarean scar disorder, retained products of conception, adenomyosis, and endometrial hyperplasia. CE is also involved in unexplained infertility, RIF in in vitro fertilization–embryo transfer cycles, and unexplained RPL. Meanwhile, the causality between CE and female infertility or pregnancy loss remains undetermined.” and reached a consensus in Round 1, as seven items in this statement received an agreement on gynecologic diseases associated with CE. These include fallopian tubal infertility (hydrosalpinx and tubal occlusion; agreement rate 74%; agree, 23/31; neutral, 8/31; disagree, 0/31) [25, 26, 27, 28], endometrial polyps (agreement rate 87%; agree, 27/31; neutral, 3/31; disagree, 1/31) [29, 30, 31, 32, 33, 34], intrauterine adhesion/Asherman syndrome (agreement rate 74%; agree, 23/31; neutral, 8/31; disagree, 0/31) [34, 35, 36, 37, 38], cesarean scar disorder (agreement rate 81%; agree, 25/31; neutral, 3/31; disagree, 3/31) [39, 40, 41], unexplained infertility (agreement rate 87%, agree, 27/31; neutral, 4/31; disagree, 0/31) [42, 43, 44], RIF (agreement rate 97%; agree, 30/31; neutral, 0/31; disagree, 1/31) [3, 45, 46, 47, 48, 49, 50, 51, 52, 53, 54, 55, 56], RPL (agreement rate 94%; agree, 29/31; neutral, 1/31; disagree, 1/31) [55, 56, 57, 58, 59, 60, 61, 62]. Meanwhile, five items that did not receive a 70% or more agreement/disagreement rate were excluded from the statement. These included endometriosis (agreement rate 65%; agree, 20/31; neutral, 6/31; disagree, 5/31) [61, 62, 63, 64], polycystic ovarian syndrome (disagreement rate 65%; agree, 5/31; neutral, 6/31; disagree, 20/31) [65], retained products of conception (agreement rate 48%; agree, 15/31; neutral, 14/31; disagree, 2/31) [66, 67], adenomyosis (agreement rate 52%; agree, 16/31; neutral, 13/31; disagree, 2/31) [68], and endometrial hyperplasia (agreement rate 45%; agree, 14/31, neutral, 10/31; disagree, 7/31) [69].

Statement 1B *Endometrial osseous metaplasia is a rare disease associated with CE and infertility*.

The statement “Endometrial osseous metaplasia is an uncommon disease associated with CE and infertility. Hysteroscopic removal of the lesions can improve the fecundity in women with endometrial osseous metaplasia.” did not reach a consensus with an agreement rate of 55% (agree, 17/31; neutral, 13/31; disagree, 1/31) in Round 1. The statement was reworded/reformulated as above and carried over to Round 2, where the revised statement received 71% agreement (agree, 22/31; neutral, 9/31; disagree, 0/31) and reached a consensus.

Endometrial osseous metaplasia (also referred to as endometrial ossification, ectopic intrauterine bone, and heterotopic intrauterine bone) is a rare clinical entity characterized by the presence of bone in the endometrium [70]. Approximately 100 cases of endometrial osseous metaplasia have been reported in the literature, and the incidence is estimated to be around 0.3/1,000. Endometrial osseous metaplasia is most frequently identified in women of reproductive age. In most cases, patients with endometrial osseous metaplasia have a history of pregnancy. Some studies suggest that endometrial osseous metaplasia results from persistent inflammation caused by necrotized nonbony embryonic tissue retained in the uterine cavity. Fertility‐sparing surgery using hysteroscopic resection/grasper or dilatation and curettage has been used for women with endometrial osseous metaplasia desiring babies. A review article described that 56% of infertile women with endometrial osseous metaplasia achieved a pregnancy [71].

Statement 1C *Several risk factors have been proposed regarding the onset of CE. A history of continuous intrauterine contraceptive device use, prolonged menstruation, atypical uterine bleeding, and pregnancy loss (miscarriage) are the independent risk factors for CE*.

This statement was reworded and reformulated from “Several risk factors have been proposed regarding the onset of CE. In women using intrauterine contraceptive devices continuously, prolonged accumulation of ESPC is often seen even after their removal from the uterine cavity. Multiple studies agree that prolonged menstruation and atypical uterine bleeding are independent risk factors for CE. Meanwhile, it remains controversial if the history of abortion, pregnancy loss, and/or caesarean section is the potential risk factor for CE.” and reached a consensus in Round 1, as four items on the independent risk factors for CE received a 70% or more agreement. These include a history of intrauterine contraceptive device use (agreement rate 71%; agree, 22/31; neutral, 9/31; disagree, 0/31) [72, 73], prolonged menstruation (agreement rate 71%; agree, 22/31; neutral, 7/31; disagree, 2/31) [74], atypical uterine bleeding (agreement rate 74%; agree, 23/31; neutral, 6/31; disagree, 2/31) [69, 74], and pregnancy loss (miscarriage, agreement rate 81%; agree, 25/31; neutral, 5/31; disagree, 1/31) [75, 76]. Meanwhile, two items that did not reach 70% or more agreement/disagreement were excluded from the statement. These include the history of artificial abortion (agreement rate 58%, agree, 18/31; neutral, 13/31; disagree, 0/31) [73] and cesarean section (agreement rate 48%; agree, 15/31; neutral, 12/31; disagree, 4/31) [74].

### 3.3 Domain 2 (Symptomatology)

Statement 2 *CE is generally asymptomatic or oligosymptomatic with subtle and nondescript gynecologic manifestations*.

The statement “CE is generally asymptomatic or oligosymptomatic with subtle and nondescript manifestations, such as vaginal spotting, abdominal discomfort, leukorrhea, intermenstrual bleeding, and prolonged menstrual bleeding.” did not reach a consensus in Round 1 regarding any items on symptoms associated with CE: vaginal spotting (agreement rate 52%; agree, 16/31; neutral, 6/31; disagree, 9/31); abdominal discomfort (agreement rate 39%; agree, 12/31; neutral, 10/31; disagree, 9/31); leukorrhea (agreement rate 39%; agree, 12/31; neutral, 11/31; disagree, 8/31), intermenstrual bleeding (agreement rate 61%; agree, 19/31; neutral, 6/31; disagree, 6/31), and prolonged menstrual bleeding (agreement rate 55%; agree, 17/31%; neutral, 6/31; disagree, 8/31). The statement was reworded/reformulated as above and carried over to Round 2, and reached a consensus with 84% agreement (agree, 26/31; neutral, 1/31; disagree, 4/31).

In contrast to acute endometritis, which is manifested with fever, pelvic pain, and vaginal discharge, the subtle and nondescript gynecologic symptoms in CE are often unnoticed by patients and even by experienced gynecologists [1, 2, 77]. It was reported that a quarter of the affected patients lack symptoms [73, 74, 75].

### 3.4 Domain 3 (Etiology and Pathogenesis)

Statement 3A *CE is a localized inflammatory disorder of the endometrium that is histopathologically characterized by superficial mucosal edema, increased endometrial stromal cell density, unsynchronized differentiation between endometrial epithelial cells and stromal cells, and unusual infiltration of endometrial stromal plasma cells. Of these histopathologic features, the most specific and sensitive findings of CE are thought to be the presence of multiple stromal plasma cells*.

This statement received 90% agreement (agree, 28/31; neutral, 3/31; disagree, 0/31) and reached a consensus in Round 1.

The history of CE dates back to the 19th century [78]. The first appearance of comprehensive research on CE was in 1907, when seminal work by Hitschman and Adler [79] adopted the infiltration of plasma cells in the endometrial stroma as the sole criterion for a diagnosis of CE and found that the presence of ESPCs is associated with pelvic inflammatory diseases, postpartum and postabortum endometritis, and retained products of conception. Since then, ESPC infiltrates have been recognized as a key feature in the diagnosis of CE. Meanwhile, a few of these cells are sporadically detectable in the “nonpathological” endometrium. In 1981, based upon their observations of 891 endometrial samples, Greenwood and Moran [1] pointed out the limitation of ESPCs alone for the histopathologic diagnosis of CE and emphasized the importance of other epithelial and stromal findings, such as superficial mucosal edema, increased stromal cell density, and unsynchronized differentiation between endometrial epithelial cells and stromal cells. ESPCs morphologically resemble endometrial stromal component cells, including macrophages and fibroblasts, and are often indistinguishable from these cells by conventional tissue staining. In 1983, Crum et al. [2] found that the combination of hematoxylin/eosin and immunoperoxidase staining effectively identifies ESPCs that are overlooked by conventional staining alone. In 1996, CD138/syndecan‐1 was discovered as a cell surface marker expressed on the plasma membrane of ESPCs [80]. Bayer‐Garner and Korourian [9] first demonstrated that immunohistochemistry with an anti‐CD138 monoclonal antibody is available and time‐saving for identifying and quantifying ESPCs in the histopathologic diagnosis of CE. They stressed that relying on the mere presence of ESPCs to diagnose CE should be avoided.

Statement 3B *The etiology and pathogenesis of CE, including its onset, progress, and remission, remain unclear. Endometrial shedding during the menstrual period is unlikely to contribute significantly to the treatment of CE. In some cases, CE seems to recur following treatment*.

This statement received 94% agreement (agree, 29/31; neutral, 2/31; disagree, 0/31) and reached a consensus in Round 1.

Not what it sounds like, the clinical course of CE remains largely unknown [81]. As a benign disease, interventional endometrial biopsy and histopathologic examinations for CE have not been favored in gynecologic practice. Accurate histopathologic diagnosis of CE has been demanding and time‐consuming until recently. In a study that employed endometrial biopsy/histopathology/immunohistochemistry (the presence of CD138^+^ ESPCs) and hysteroscopy (the findings proposed by the International Working Group for Standardization of Chronic Endometritis Diagnosis) in the proliferative phase, 128 women were diagnosed with CE. Of them, 64 women with CE were followed up for two complete menstrual cycles without antibiotic treatment and reexamined for CE. After two menstrual periods, CE persisted in 60 women (cure rate 6.25%) [82]. In another study, using endometrial biopsy/histopathology/immunohistochemistry and hysteroscopy in the proliferative phase, 32 infertile women were diagnosed with CE and monitored without antibiotic treatment up to 6 months. Persistent CE was detected in all 32 women [83]. These findings suggest that endometrial shedding during the menstrual period is unlikely to contribute to the remission or recovery of CE. In a case–control study that tracked 105 women who overcame CE but experienced pregnancy loss or failed to conceive for a maximum of 18 months in the following infertility treatment, the recurrence of CE was detected histopathologically and bacteriologically in 29.5% (31/105) of these women. A history of hysteroscopic surgery was associated with a lower risk of CE recurrence, whereas a history of pregnancy loss was associated with an increased risk. While the cumulative CE recurrence rates in 49 patients without a history of pregnancy loss were 5.6% at 6 months, 13.5% at 12 months, and 20.4% at 18 months, those in 56 patients with a history of pregnancy loss were 47.1% at 6 months, 36.2% at 12 months, and 37.5% at 18 months [76].

Statement 3C *Chronic deciduitis is potentially a persistent form of CE during pregnancy and is associated with preterm labor*.

This statement was reworded and reformulated from “Chronic deciduitis is potentially a persistent form of CE during pregnancy. Chronic deciduitis is associated with obstetric/neonatal complications, such as preterm labor, preeclampsia, periventricular leukomalacia, and cerebral palsy in premature infants.” and reached a consensus in Round 1, as only one item in this statement, preterm labor (agreement rate 71%; agree, 22/31; neutral, 9/31; disagree, 0/31) received an agreement as an obstetric complication. Meanwhile, three items including preeclampsia (agreement rate 55%; agree, 17/31; neutral, 11/31; disagree, 3/31), periventricular leukomalacia (agreement rate 26%; agree, 8/31; neutral, 17/31; disagree, 6/31), and cerebral palsy (agreement rate 16%; agree, 5/31; neutral, 16/31; disagree, 10/31) were excluded from the statement.

Chronic deciduitis is a pathological inflammatory condition in the decidualized endometrium [84]. Like CE, chronic deciduitis is histopathologically recognized as an unusual infiltration of ESPCs during pregnancy. The causality between CE and chronic deciduitis remains unclear. One study reported that a cluster or number of decidual stromal plasma cells was frequently found when women with untreated CE conceived but resulted in pregnancy loss, suggesting a potential continuity between CE and chronic deciduitis [85].

Statement 3D *One of the major causes of CE is microbial infection in the uterine cavity. This is supported by the fact that some antibiotic therapies are effective in eliminating endometrial stromal plasma cells in women with CE*.

This statement received 90% agreement (agree, 28/31; neutral, 2/31; disagree, 1/31) and reached a consensus in Round 1.

One of the major causes of CE is microbial infection in the uterine cavity. Microbial antigens, such as lipopolysaccharide, potentially trigger immune responses unusual to the human endometrium by inducing the molecules associated with B cell extravasation, migration, and differentiation [C‐X‐C motif chemokine ligand (CXCL) 13, CD62E, CXCL1, interleukin (IL)‐6, IL‐1, and tumor necrosis factor‐] [42, 86]. These abnormal local microenvironments in the uterine cavity potentially affect the expression of the molecules associated with endometrial proliferation [B‐cell/CLL lymphoma 2 (BCL2), Bcl‐2‐associated X protein, Ki‐67, estrogen receptor‐, and ‐], endometrial receptivity (progesterone receptor, IL‐11, C‐C motif chemokine ligand 4, insulin‐like growth factor (IGF)‐1, and caspase‐8), and endometrial decidualization (prolactin and IGF binding protein‐1) and pose a negative effect on the embryo implantation process [87, 88]. Some antibiotic therapies are effective in eliminating ESPCs in patients with CE [3].

### 3.5 Domain 4 (Microbiology)

Statement 4A *The microorganisms detected in the uterine cavity of women with CE include common bacteria, such as Streptococcus, Escherichia coli, Enterococcus faecalis, and Staphylococcus species, as well as Mycoplasma/Ureaplasma species*.

This statement was reworded and reformulated from “The microorganisms detected in the uterine cavity of women with CE include common bacteria, such as *Streptococcus*, *Escherichia coli* , *Enterococcus faecalis* , and *Staphylococcus* species, as well as *Mycoplasma*/*Ureaplasma* species, *Proteus* species, *Klebsiella pneumoniae* , *Pseudomonas aeruginosa* , *Gardnerella vaginalis* , *Corynebacterium*, and yeasts.” and reached a consensus in Round 1, as six items on the microorganisms detected in the uterine cavity of women with CE received a 70% or more agreement. These include *Streptococcus* (agreement rate 74%; agree, 23/31; neutral, 7/31; disagree, 1/31), *Escherichia coli* (agreement rate 77%; agree, 24/31; neutral, 6/31; disagree, 1/31), *Enterococcus faecalis* (agreement rate 71%; agree, 22/31; neutral, 7/31; disagree, 2/31), *Staphylococcus* species (agreement rate 71%; agree, 22/31; neutral, 6/31; disagree, 3/31), *Mycoplasma* species (agreement rate 74%; agree, 23/31; neutral, 7/31; disagree, 1/31), and *Ureaplasma* species (agreement rate 77%; agree, 24/31; neutral, 6/31; disagree, 1/31). Meanwhile, six items that did not reach 70% or more agreement/disagreement rate were excluded from the statement. These were proteus species (agreement rate 52%; agree, 16/31; neutral, 14/31; disagree, 1/31), *Klebsiella pneumoniae* (agreement rate 61%; agree, 19/31; neutral, 10/31; disagree, 2/31), *Pseudomonas aeruginosa* (agreement rate 45%; agree, 14/31; neutral, 12/31; disagree, 5/31), *Gardnerella vaginalis* (agreement rate 65%; agree, 20/31 neutral, 8/31; disagree, 3/31), *Corynebacterium* (neutral rate 52%; agree, 13/31; neutral, 16/31; disagree, 2/31), and yeasts (neutral rate 48%; agree, 6/31; neutral, 15/31; disagree, 10/31).

In a comprehensive study using conventional culture and immunoassay, the most frequently detected infectious agents in the endometrium were common bacteria, which account for 58% of all cases, followed by *Ureaplasma urealyticum* (10%). While *Streptococci* were found in 27.9%, the intestinal bacterial species ( *Enterococcus faecalis* and *Escherichia coli* ) were detected in 25.5% of cases [89]. Notably, in only 32.6% of cases, the same bacteria were isolated from both endometrial and vaginal cultures, suggesting a low reliability of conventional vaginal culture in predicting the presence of CE [89]. Meanwhile, the detection rates of *Chlamydia trachomatis* (2.7%) and *Neisseria gonorrhoeae* (0%), the principal pathogens causing acute endometritis [90, 91], are low in women with CE [89]. In addition, the administration of azithromycin or cefixime, the antibiotics targeting *Chlamydia trachomatis* and *Neisseria gonorrhoeae* , was reported to have failed to preserve future fertility in women with CE [92].

Antibiotic treatment can effectively reduce ESPC density in women with CE, but not in all cases, suggesting a potential involvement of pathogens other than bacteria in the onset of CE. Several case reports described the presence of herpes simplex virus, cytomegalovirus, and human immunodeficiency virus in the endometrium with ESPC infiltrates [93].

Statement 4B *Mycobacterium tuberculosis is a microorganism that causes chronic granulomatous endometritis, a subtype of CE characterized by poorly developed caseating granuloma and surrounding lymphocyte infiltrates, including stromal plasma cells*.

This statement received 74% agreement (agree, 23/31; neutral, 8/31; disagree, 0/31) and reached a consensus in Round 1.

*Mycobacterium tuberculosis* is a microorganism that causes female genital tuberculosis and subsequent infertility, which is a serious medical problem in certain endemic areas in the world [94, 95]. Poorly developed caseating granulomas with surrounding lymphocyte infiltrates, including ESPCs, are a histopathologic hallmark of CE associated with *Mycobacterium tuberculosis* infection [96]. Polymerase chain reaction tests using endometrial biopsy samples are widely used to detect *Mycobacterium tuberculosis* [97, 98]. Antitubercular chemotherapy, including isoniazid (300 mg per day), rifampicin (450–600 mg per day), ethambutol (800–1,200 mg per day), and pyrazinamide (1,200–1,500 mg per day), is effective for infertile women with chronic granulomatous endometritis. After 6 months of antitubercular treatment, the clinical pregnancy rate within 12 months was reported to be about 90% [96].

### 3.6 Domain 5 (Diagnosis)

Statement 5A *Endometrial biopsies are recommended to obtain the specimens for histopathologic diagnosis of CE in infertile women. Endometrial samplings by smear or brushing are not recommended*.

This statement received 94% agreement (agree, 29/31; neutral, 0/31; disagree, 2/31) and reached a consensus in Round 1.

Unlike acute endometritis, which is characterized by microabscess formation and neutrophil invasion in the uterine cavity, endometrial glandular lumina, and superficial layers, the main inflammatory lesions in CE are located in the endometrial stromal areas, occasionally in the deeper functional layers and basal layers. Endometrial biopsies are indispensable for histopathologic diagnosis of CE [99, 100]. Endometrial samples obtained by smear or brushing are not suitable for this purpose. Few studies investigated the endometrial sampling methods. A prospective cross‐sectional study with a small sample size (*n* = 40) failed to demonstrate any statistical differences between pipelle biopsy and curettage in the diagnostic accuracy of CE in patients with RIF [101]. Pipelle biopsy may be more cost‐effective and less complicated compared with curettage. ESPCs tend to accumulate focally in the endometrial stromal compartments rather than distributing evenly; thus, ESPCs may be missed in small biopsy specimens [49]. In some women with CE, ESPCs amass only in the endometrial basal layer [73]. Although examinations using the tissues obtained via “whole‐wall” endometrial curettage may raise the possibility of detecting ESPCs, this sampling method can be harmful to women desiring pregnancy, causing complications such as endometrial thinning and intrauterine adhesions/Asherman's syndrome, which deteriorate their reproductive outcomes [13].

Statement 5B *The addition of immunohistochemistry for CD138, a plasma cell marker, improves sensitivity, specificity, interobserver variability, and intraobserver variability compared with the conventional tissue staining alone in the histopathologic diagnosis of CE*.

This statement received 90% agreement (agree, 28/31; neutral, 1/31; disagree, 2/31) and reached a consensus in Round 1.

With conventional tissue staining, identification of ESPCs is not easy even for experienced pathologists [11]. Typical plasma cells are described as cells with a large cell body, a high nuclei/cytoplasm ratio, basophilic cytoplasm, and nuclei with heterochromatin rearrangement referred to as the “spoke‐wheel” or “clock‐face” patterns. Under microscopic examination, these morphological features are not always evident in ESPCs, as they often exhibit an appearance similar to stromal fibroblasts and mononuclear leukocytes. Some histological findings common in the secretory phase endometrium, such as superficial edematous change and elevated stromal cell density, can also interfere with the identification of ESPCs. Detection of glandular‐stromal dyssynchrony and eosinophil infiltrates (cytoplasmic eosinophilic granules) in the conventionally stained endometrial sections was proposed as a convenient screening tool to predict the presence of ESPCs, but it is not an absolute finding in CE. Histopathologic evaluation using immunohistochemistry for plasmacyte marker CD138 (syndecan‐1, a transmembrane‐type heparan sulfate proteoglycan) is currently the most reliable and time‐saving diagnostic method for CE [102]. Immunostaining for CD138 was found to be superior in the detection of ESPCs to conventional tissue staining with methyl green pyronin, hematoxylin, and eosin (odds ratio, 2.8; sensitivity, 100% vs. 75%; specificity, 100% vs. 65%) with less interobserver (96% vs. 68%) and intraobserver variability (93% vs. 47%) [10, 11].

Statement 5C *Optimized laboratory test settings and quality control of CD138 immunostaining, including antibody selection and dilution, incubation time, thickness of endometrial sections, and number and area of sections, are indispensable for accurate diagnosis of CE*.

This statement received 94% agreement (agree, 29/31; neutral, 2/31; disagree, 0/31) and reached a consensus in Round 1.

An optimal setting in clinical examinations is essential for the accurate evaluation of ESPCs. Standardized techniques and conditions, however, remain to be established for immunohistochemical identification of ESPCs [13]. The histopathologic diagnosis of CE is potentially influenced by multiple laboratory factors, including the types of primary antibodies, the conditions for secondary detection systems, and the thickness, area, and number of fields and/or sections observed in the detection of ESPCs. Indeed, studies suggest that the dilution of the primary antibodies may have an impact on the histopathologic diagnostic rates of CE in infertile women. For example, one study using a 1:1,000 dilution of clone B‐B4, an anti‐CD138 monoclonal antibody, showed that the prevalence of CE was 2.8% in asymptomatic infertile women undergoing their first in vitro fertilization‐embryo transfer attempt [45]. Meanwhile, another study using a higher concentration (1:100 dilution) of B‐B4 identified CE in 12%–30% of infertile patients [3, 42]. Nordic immunohistochemical Quality Control (NordiQC) is a professional scientific organization of pathologists for promoting the quality of immunohistochemistry and expanding its clinical use by arranging schemes for immunohistochemical proficiency testing and providing examples of recommended protocols, tissue controls, and other relevant information, including descriptions of epitopes and technical protocol parameters. On their website (https://www.nordiqc.org/epitope.php?id=37), NordiQC provides the assessment results and recommended protocols of immunohistochemistry for CD138 [103].

Statement 5D *Caution should be exercised in the interpretation of the results of immunohistochemistry in CE, as not only endometrial stromal plasma cells but endometrial epithelial cells constitutively express CD138. The conditions of the section preparation and/or staining intensity may cause a mix‐up between these two cell types, potentially leading to the overdiagnosis of CE*.

This statement received 81% agreement (agree, 25/31; neutral, 5/31; disagree, 1/31) and reached a consensus in Round 1.

The results of CD138 immunostaining should be interpreted with caution. Endometrial surface/glandular epithelial cells constitutively express CD138 mainly on the basolateral sides of their plasma membrane [104, 105]. Many of the primary antibodies targeting CD138 on ESPCs also recognize the epitope of this antigen expressed on endometrial epithelial cells, although the staining intensity is generally weaker than that in ESPCs [102, 105, 106]. The conditions of the sections and immunostaining may misidentify epithelial cells as ESPCs, leading to potential overdiagnosis of CE. Combining immunohistochemistry and conventional nucleic staining is recommended to avoid this kind of misinterpretation. Another potential marker for ESPC is multiple myeloma oncogene (MUM)‐1 (also known as interferon regulatory factor 4), a transcription factor expressed in the late plasma cell‐directed stages of differentiating B cells [107]. It was demonstrated that the sensitivity, specificity, and overall diagnostic accuracy of MUM‐1 in the immunohistochemical detection of ESPCs were similar to those of CD138 [108]. Meanwhile, in women with CE, MUM‐1 identified a higher number of ESPCs and showed a higher inter‐observer agreement compared with CD138. While the advantage of MUM‐1 over CD138 is that this molecular marker is not expressed on endometrial surface/glandular epithelial cells, its drawback is that it is expressed on activated T cells [109], as human endometrium contains substantial numbers of activated T cell subpopulations [110, 111]. Dual immunostaining for CD138 and MUM‐1 potentially makes up for the shortcomings of each molecular marker in the detection of ESPC [112].

Statement 5E *The detection rate of endometrial stromal plasma cells in CE is higher in the proliferative phase of the menstrual cycle than in the secretory phase*.

This statement received 81% agreement (agree, 25/31; neutral, 4/31; disagree, 2/31) and reached a consensus in Round 1.

Studies generally agree that, in CE, the detection rate and density of ESPCs are higher in the proliferative phase of the menstrual cycle than in the secretory phase [69, 113, 114]. These findings were strengthened by a recent study that compared the density of ESPCs between the proliferative phase and secretory phase in identical menstrual cycles in infertile women [83]. Another study demonstrated that the detection rate of ESPCs in CE is higher in the early proliferative phase of the menstrual cycle than in the late proliferative phase [115]. The results of histopathological examinations should be detailed and optimized according to the date when the endometrial biopsy was performed in the menstrual cycle.

Statement 5F *The solid threshold of endometrial stromal plasma cell density and the number of microscopic fields to be observed for identification of endometrial stromal plasma cells remain undetermined for histopathologic diagnosis of CE. Given the reproductive outcomes in the following infertility treatment cycles, a threshold of ≥ 5 plasma cells/10 high power fields (≥ 0.5 plasma cells/high power field) may be appropriate for the histopathologic diagnosis of CE*.

This statement was reworded and reformulated from “The solid threshold of endometrial stromal plasma cell density and the number of microscopic fields to be observed for identification of endometrial stromal plasma cells remain undetermined for histopathologic diagnosis of CE. Given the reproductive outcomes in the following infertility treatment cycles, ≥ 5 endometrial stromal plasma cells/high power field can be histopathologically diagnosed as CE and may be eligible for antibiotic treatment. A threshold of ≥ 5 plasma cells/10 high power fields (≥ 0.5 plasma cells/high power field) may be more appropriate for the histopathologic diagnosis of CE.” While the threshold of ≥ 5 endometrial stromal plasma cells/high power field received an agreement of 58% (agree, 18/31; neutral, 7/31; disagree, 6/31), the threshold of ≥ 5 endometrial stromal plasma cells/10 high power fields (≥ 0.5 plasma cells/high power field) received an agreement of 81% (agree, 25/31; neutral, 1/31; disagree, 5/31) in Round 1.

To date, standardized assessment methods for the solid threshold for ESPC density and the number of microscopic fields to be observed for identification of these cells are conspicuously lacking in the histopathologic diagnosis of CE. Several thresholds for ESPC density have been proposed in the literature. While some authors claim that identification of a single ESPC within an endometrial biopsy sample is sufficient for the histopathologic diagnosis of CE, others state that the infiltration of multiple ESPCs is a requirement for the histopathologic diagnosis of CE.

In a meta‐analysis/systematic review that included a total of 4145 patients from ten studies, infertile women with CE had a lower ongoing pregnancy rate/live birth rate (odds ratio 1.97, *p* = 0.02) and clinical pregnancy rate (odds ratio 2.28, *p* = 0.002) compared to those without CE [116]. The cure of CE following antibiotic treatment increased the reproductive outcomes in these women to the level of infertile women without CE [ongoing pregnancy/live birth rate (odds ratio 5.33, *p* < 0.0001) and clinical pregnancy rate (odds ratio 3.64, *p* = 0.0001)]. Women with ≥ 5 ESPCs/high power field (HPF) had lower ongoing pregnancy/live birth rate (odds ratio 0.43, *p* = 0.003) and clinical pregnancy rate (odds ratio 0.40, *p* = 0.0007) compared to those with 1–4 ESPCs/HPF. Meanwhile, the pregnancy outcomes in subsequent embryo transfer cycles were similar between infertile women with 1–4 ESPCs/HPF and those without detectable ESPCs.

In another meta‐analysis/systematic review that included nine studies adopting different thresholds (1 to 50 ESPCs/10 HPFs) for histopathologic diagnosis of CE, a significant association was found between 0.5 ≥ ESPC/HPF and miscarriage rate (risk ratio 2.4; *p* = 0.007). A threshold of 5 ≥ ESPCs/10 HPFs (0.5 ≥ ESPC/HPF) may be more appropriate for the diagnosis of CE [117]. The diagnosis of CE in patients with low or borderline ESPC values (< 5 ESPCs/10 HPFs) should be integrated with clinical history and associated hysteroscopic findings, including strawberry aspect, focal hyperemia, hemorrhagic spots, micropolyps, and edema in the endometrium. In patients with fewer than 5 ESPCs/10 HPFs (0.5 ESPC/HPF), the requirement for antibiotic treatment should be judged on a case‐by‐case basis. Note that different diagnostic criteria should be adopted depending on the timing (proliferative phase or secretory phase) of endometrial sampling.

Statement 5G *Fluid hysteroscopy emerges as an alternate minimally invasive diagnostic modality for CE. Five hysteroscopic features suggestive of CE are proposed by the Delphi consensus method. These include (1) strawberry aspect: large areas of hyperemic endometrium flushed with white central points, (2) focal hyperemia: small areas of hyperemic endometrium, (3) hemorrhagic spots: focal red areas with sharp and irregular borders possibly in continuity with capillary, (4) endometrial micropolyps: small intrauterine new growths < 1 mm in size with a distinct connective vascular axis, distributed on focal areas or the entire endometrial surface, and (5) endometrial edema in the follicular phase: thick and pale appearance of the endometrium owing to stromal edema (a normal finding during the secretory phase)*.

All items in this statement reached a consensus in Round 1, including strawberry aspect (agreement rate 97%; agree, 30/31; neutral, 1/31; disagree, 0/31) [15, 118, 119], focal hyperemia (agreement rate 84%; agree, 26/31; neutral, 5/31; disagree, 0/31) [118, 119], hemorrhagic spots (agreement rate 81%; agree, 25/31; neutral, 6/31; disagree, 0/31) [118, 119], endometrial micropolyps (agreement rate 94%; agree, 29/31; neutral, 2/31; disagree, 0/31) [14, 118, 119, 120] and endometrial edemas (agreement rate 71%, agree, 22/31; neutral, 9/31; disagree, 0/31) [118, 119].

In a 2019 Delphi consensus study by the International Working Group for Standardization of Chronic Endometritis Diagnosis, these five findings were proposed as hysteroscopic features suggestive of CE [118].

Statement 5H *There is substantial incompatibility between histopathologic and hysteroscopic diagnosis of CE. In the current clinical practice, fluid hysteroscopy alone is insufficient for the accurate diagnosis of CE*.

This statement received 74% agreement (agree, 23/31; neutral, 5/31; disagree, 3/31) and reached a consensus in Round 1.

Fluid hysteroscopy is generally reported to be a highly sensitive diagnostic tool for conventional intrauterine lesions, with an accuracy ranging from 85% to 98% [121]. Meanwhile, studies estimate the sensitivity and specificity of fluid hysteroscopy in predicting histopathologic CE as 35%–65% and 66%–99%, respectively, with an accuracy ranging from 65% to 90% [14, 15, 16, 17, 18]. Rather, lower sensitivity and higher specificity suggest that the presence of hysteroscopic CE findings predicts the presence of histopathologic CE. In contrast, the absence of hysteroscopic CE findings cannot deny the presence of histopathologic CE. Fluid hysteroscopy is a promising office procedure for the diagnosis of CE with less invasiveness and wider‐range observation than the histopathologic diagnosis of CE using endometrial biopsy samples. More studies are warranted to evaluate the usefulness of hysteroscopy in diagnosing CE.

Statement 5I *Recent progress in molecular microbiology (next‐generation sequencing testing of the 16S rRNA targeted gene or real‐time polymerase chain reaction for the causal pathogens of CE) enabled the comprehensive analysis of culturable and non‐culturable pathogens associated with CE*.

This statement received 77% agreement (agree, 24/31; neutral, 7/31; disagree, 00/31) and reached a consensus in Round 1.

The human uterine cavity has long been considered to be germ‐free. High‐throughput techniques using next‐generation sequencing analysis of the 16S rRNA gene, however, demonstrated the presence of microbiota in the human endometrium and uterine cavity [122]. Contrary to the literature agreeing that *Lactobacillus*‐dominant microbiota (LDM, *Lactobacillus* composition of 90% or more, particularly *L. crispatus* ) is the representative vaginal microbiota in healthy premenopausal women, the definition of the normal endometrial microbiota remains controversial, as the estimated bacterial load in the uterine cavity is considered to be 1/100 to 1/10,000‐fold fewer than in the vaginal cavity [123]. While an association between shifting to a non‐LDM profile (< 90% *Lactobacillus*) in the endometrial microbiota and low live birth rate (6.7% in non‐LDM vs. 58.8% in LDM) was demonstrated in embryo transfer cycles [124, 125], subsequent studies failed to find a negative impact of non‐LDM endometrial microbiota on pregnancy outcomes [126, 127]. In addition, another study reported that the proportion of *Lactobacillus* was similar between fertile women and infertile women with a history of RIF (55%–60%) [128].

Meanwhile, a 16S rRNA gene‐targeted real‐time polymerase chain reaction test was developed to detect nine CE‐associated culturable and non‐culturable pathogens, including *Chlamydia trachomatis* , *Enterococcus*, *Escherichia coli* , *Gardnerella vaginalis* , *Klebsiella pneumoniae* , *Mycoplasma hominis* , *Neisseria gonorrhoeae* , *Staphylococcus*, and *Streptococcu*s (analysis of infectious CE, ALICE) [129]. The individual matching accuracy between the test and histopathologic, hysteroscopic, and microbial culture diagnoses was 46.15%, 58.46%, and 56.92%, respectively, but that increased to 76.92% (75% sensitivity, 100% specificity, 100% positive predictive value, 25% negative predictive value, 0% false‐positive, and 25% false‐negative rates) and reached a similar level to all three classic diagnostic methods were combined [130]. A prospective multicenter cohort study showed that the ALICE‐guided antibiotic treatment strategy against CE improves the reproductive outcomes (clinical pregnancy rate and ongoing pregnancy rate) in women with a history of RIF and RPL [130].

Further studies are needed to assess the diagnostic accuracy of molecular microbiology tests for CE.

### 3.7 Domain 6 (Treatment)

Statement 6A *If CE is diagnosed in women with a history of recurrent implantation failure, antibiotic treatment can improve subsequent pregnancy outcomes*.

This statement received 84% agreement (agree, 26/31; neutral, 3/31; disagree, 2/31) and reached a consensus in Round 1.

A randomized controlled study in 2011 demonstrated that CE is rarely diagnosed (2.8%, 17 out of 623) in asymptomatic infertile women with a normal transvaginal ultrasound who underwent hysteroscopy‐guided endometrial biopsy and immunohistochemistry before their first in vitro fertilization/intracytoplasmic sperm injection attempt [45]. The cumulative live birth rate (including spontaneous pregnancies) was at a similar level between patients with or without CE (76% vs. 54%), as well as the clinical pregnancy rate per embryo transfer (hazard ratio 1.456, 95% confidence interval 0.770–2.750), indicating that the negative effect of CE on this cohort of infertile women is minimal. Meanwhile, in a 2010 retrospective study, CE was immunohistochemically identified in 10 out of 33 (30.3%) of infertile women with a history of RIF [3]. Following successful antibiotic treatment (100%) with 14‐day oral doxycycline (100 mg twice per day) and additional 14‐day ciprofloxacin and metronidazole (500 mg twice per day, respectively) for persistent CE, the clinical pregnancy rate or ongoing pregnancy rate in the subsequent embryo transfer cycles of these women were similar to those with a history of RIF but without CE or those not undergoing examinations for CE. These findings suggest that CE is a contributing factor to RIF. Thereafter, accumulating studies reported an association between CE and RIF, the therapeutic potential of antibiotic treatment to eradicate ESPCs in CE, and improve reproductive outcomes in subsequent embryo transfer cycles following treatment [4, 46, 131, 132]. In a meta‐analysis including five studies, 796 patients, women receiving antibiotic treatment but without the histologic confirmation of CE cure did not show any advantage in reproductive outcome compared with untreated controls [5]. By contrast, patients with cured CE showed a higher ongoing pregnancy rate/live birth rate [odds ratio (OR): 6.81, 95% confidence interval (CI): 2.08–22.24, *I* ^2^ = 0%, *p* = 0.001], clinical pregnancy rate (OR 4.98, 95% CI 1.72–14.43, *I* ^2^ = 0%, *p* = 0.003), and implantation rate (OR 3.24, 95% CI 1.33–7.88, *I* ^2^ = 0%, *p* = 0.01) in comparison with those with persistent CE, whereas the miscarriage rate was not different between the two groups. These findings were supported by a recent study that evaluated the risk factors for RIF defined by the European Society for Human Reproduction and Embryology and pointed out CE as the second strongest predictor for RIF [133]. Despite the encouraging results from observational cohort studies suggesting the effectiveness of antibiotic treatment in the cure of CE and improvement in the reproductive outcome in women with a history of RIF, its impact should be confirmed by randomized controlled trials, which are few in this field [134].

Statement 6B *If CE is diagnosed in women with a history of recurrent pregnancy loss, antibiotic treatment can improve subsequent pregnancy outcomes*.

This statement received 71% agreement (agree, 22/31; neutral, 8/31; disagree, 1/31) and reached a consensus in Round 1.

The prevalence of CE in women with a history of RPL, defined as three or more consecutive losses of intrauterine pregnancies before 22 weeks of gestation, has been reported as 9.3%–67.6% [30, 55, 56, 57, 60, 135, 136]. In a meta‐analysis, including eight studies, 835 participants, a higher proportion of CE was observed among women with RPL compared to controls (184 out of 489, 37.6%, vs. 57 out of 346, 16.4%), OR 3.59, 95% CI 2.46–5.24 [137]. As with RIF, a few studies, including prospective observational cohort designs, have demonstrated the effectiveness of antibiotic treatment in curing CE and potentially improving reproductive outcomes in women with cured CE undergoing RPL [57, 138]. Despite these results, its impact should be confirmed by randomized controlled trials.

Statement 6C *Surgical treatment may be prioritized in CE that is concomitant with endometrial polyps, intrauterine adhesions/Asherman syndrome, and hydrosalpinx*.

This statement was reworded and reformulated from “Surgical treatment may be prioritized in CE that is concomitant with endometrial polyps, intrauterine adhesions, hydrosalpinx, and cesarean section disorder.” and reached a consensus in Round 1, as three items regarding the female reproductive disorders associated with CE received 70% or more agreement. These included endometrial polyps (agreement rate 87%; agree, 27/31; neutral, 1/31; disagree, 3/31) [31, 33, 34], intrauterine adhesions (agreement rate 87%; agree, 27/31; neutral, 3/31; disagree, 1/31) [34], and hydrosalpinx (agreement rate 81%; agree, 25/31; neutral, 4/31; disagree, 2/31) [27]. Cesarean section disorder did not reach consensus (agreement rate: 65%; agree, 20/31; neutral, 8/31; disagree, 3/31) and was excluded from the statement.

CE often coexists with other female reproductive tract disorders, including endometrial polyps, intrauterine adhesions, hydrosalpinx, and cesarean section disorder. While hysteroscopic polypectomy alone is reported to improve ESPC infiltration in CE in 89.7% of infertile women without preemptive and/or concurrent antibiotic treatment, hysteroscopic adhesiolysis alone improves it in 92.8% of the affected women [34]. In the remaining cases of persistent CE following hysteroscopic polypectomy, additional antibiotic treatment is reported to be effective in eradicating ESPCs [35, 36]. Laparoscopic salpingostomy and/or fimbrioplasty and proximal tubal occlusion are reported to improve CE in 56.5% of infertile women with hydrosalpinx/peritubal adhesions following one menstrual cycle and 100% within 6 months [27].

Statement 6D *Antibiotic resistance is a serious global problem in the treatment of infectious diseases. Surveillance of emerging multidrug‐resistant CE is necessary*.

The statement “Antibiotic resistance is a serious global problem in the treatment of infectious diseases. Multidrug‐resistant CE is unexceptionally increasing.” received an agreement of 61% (agree, 19/31; neutral, 11/31; disagree, 1/31) and did not reach a consensus in Round 1. The statement was reworded/reformulated as above and carried over to Round 2, where the revised statement received 94% agreement (agree, 29/31; neutral, 2/31; disagree, 0/31) and reached a consensus.

In 2008, according to the results of the endometrial histopathology, culture, and antibiogram, less than 20% of CE was estimated to be resistant to single‐course oral doxycycline treatment [89]. In 2015, the same research group reported that 72% of CE exhibited resistance to single‐course antibiogram‐oriented antibiotic regimens based on Centers for Disease Control guidelines. Furthermore, 50% were resistant to the second course (the repeat of the first course regimen) of treatment, and 25% were resistant to the third course [4]. Using a stringent criterion for CE (5 ≥ ESPCs/HPF), another research group reported that 25% of CE were resistant to the single course of treatment with a combination of levofloxacin lactate (200 mg orally twice a day) plus metronidazole (500 mg orally 3 times a day) for 14 days [139]. Moreover, 11.0% of CE was resistant to the second course (the repeat of the first course) of treatment. A more recent cross‐sectional study conducted between 2020 and 2024 reported an increasing trend of antibiotic‐resistant CE over time. For example, ampicillin resistance of *Enterococcus* reached 98.5% (63/64), penicillin resistance of *Streptococcus agalactiae* and *Streptococcus bovis* was 30.8% (16/52), and resistance to both penicillins and cephalosporins/extended‐spectrum beta‐lactamase positivity of *Escherichia coli* and *Klebsiella* was 34.7% (25/72) (*p* < 0.001). Resistance to commonly used first‐line empiric oral antibiotics against CE, such as tetracyclines (75.8%, 185/244), quinolones (68.4%, 167/244), metronidazole (39.3%, 96/244), and clindamycin (50.4%, 123/244), was also substantial, whereas clarithromycin resistance remained low across the entire cohort (2.9%, 7/244) [140].

The reports on multidrug‐resistant CE are also emerging. From 2010 to 2020, antibiotic resistance in CE was tracked in more than 1000 infertile women with a history of RIF over a decade in a longitudinal study. While 21% were resistant to single doxycycline treatment (100 mg orally twice a day for 14 days), 7.8% were resistant to doxycycline and the second‐line combination of metronidazole (250 mg orally twice a day for 14 days)/ciprofloxacin (200 mg orally twice a day for 14 days). Multidrug‐resistant CE markedly increased (OR 8.27, 95% CI 2.58–26.43, *p* trend < 0.001) from the first 5 years (1.3%, April 2010–March 2015) to the last 5 years (9.6%, April 2015–March 2020) [141]. Meanwhile, another study identified multidrug resistance in 5.5% of CE cases following 2–5 cycles of antibiotic treatment, including oral doxycycline, metronidazole, and ciprofloxacin between 2019 and 2021 [142]. These findings underscore the requirement for improved and targeted diagnostic and therapeutic strategies to manage CE effectively. To prevent further antibiotic resistance, antibiogram tests may be considered in cases with intractable and refractory CE. The roles of prophylactic nutritional interventions in CE, such as proper diet with low fats and rich folates, antioxidants, and vitamins A, C, D, and E, as well as probiotic and prebiotic supplementation, are worth pursuing [143].

## 4 Discussion

This e‐Delphi consensus study provides real‐world clinical perspectives from a diverse group of international experts on the literature‐based statements regarding the clinical implications of CE.

### 4.1 Strengths

This study has several strengths. First, it contains the first comprehensive literature review on CE that covers a wide variety of current areas of interest and clinical challenges, ranging from symptomatology, etiology, pathogenesis, risk factors, diagnosis, to overall relevance on female reproductive failure (recurrent implantation failure, recurrent pregnancy loss, and obstetric complications) and management of affected infertile women. The consensus method enabled the inclusion of more topics than would typically be addressed in existing randomized controlled trials and systematic reviews/meta‐analyses [21]. Second, the consensus statements were extracted and refined by panelists over months of online web meetings, which helped ensure the objectivity of this study. Third, it benefited from the knowledge and experience of active international experts who published multiple clinical practice articles within 10 years or at least one within 3 years. The participants also specialize in infertility diagnosis and treatment and joined from across the globe, including Europe, North America, the Middle East, and Asia, which potentially reflects the quality of healthcare provision in reproductive medicine in different parts of the world.

While 11 items in nine statements received an agreement rate of 90% or more, 14 items in nine statements received an agreement rate between 80% and 90%. Notably, the threshold of ≥ 5 ESPCs/10 HPFs (≥ 0.5 ESPC/HPF) received a worldwide agreement of 81% under such circumstances (Statement 5F). This statement was derived from a meta‐analysis/systematic review that included nine studies using different thresholds (1–50 ESPCs/10 HPFs) for histopathologic diagnosis of CE [117]. This value may serve as a useful benchmark for future clinical trials on CE.

### 4.2 Limitations

This study also has some limitations; for example, there is a potential risk of missing relevant studies in the literature search. The consensus statements only represent the collective expert opinion of the participants. No statements reached unanimity. The geographic distribution of the participants appears skewed toward East Asia, particularly China and Japan, suggesting a concentration of researchers in this region. This may affect the generalizability of the results. There are potential language biases as non‐English studies were excluded. The intra‐ and inter‐observer variabilities, staining protocols, and laboratory/pathology practices may affect the reproducibility of the histopathologic diagnosis of CE. The threshold of ≥ 5 ESPCs/10 HPFs (≥ 0.5 ESPC/HPF) does not represent an evidence‐based definitive criterion, but a provisional consensus. Thus, it requires external validation. Given the consensus statements, individual patient characteristics should always be taken into consideration in clinical practice.

### 4.3 Conclusions

This e‐Delphi study provides international expert opinions on CE from a broad range of global regions. The consensus statements achieved a good level of agreement on clinically important aspects of CE, including epidemiology, symptomatology, etiology and pathogenesis, microbiology, diagnosis, and treatment. Although this study does not conclusively define CE, these consensus statements represent a structured summary of current expert perspectives and potentially serve as a reference framework for future validation studies and guideline development.

## Funding

This study was granted by the Japan Society for Reproductive Medicine.

## Ethics Statement

These statements and comments were based on existing evidence in the literature, and institutional approval on ethics was not required.

## Conflicts of Interest

The authors declare no conflicts of interest.

## Data Availability

The data that support the findings of this study (Delphi questionnaires) are available on request from the corresponding author.
